# Exploring the Acceptability, Appropriateness, and Utility of a Digital Single-Session Intervention (Project SOLVE-NZ) for Adolescent Mental Health in New Zealand: Interview Study Among Students and Teachers

**DOI:** 10.2196/81259

**Published:** 2026-01-13

**Authors:** Morgan Taylor Blind, Nicola Starkey, Amy Bird, Hoana McMillan

**Affiliations:** 1 University of Waikato Hamilton New Zealand; 2 University of Waikato Hamilton [Tauranga Campus] New Zealand

**Keywords:** digital single-session intervention, mental health, adolescent, co-design, qualitative methods, problem-solving skills

## Abstract

**Background:**

Globally, we face a significant treatment gap in mental health care, with extensive wait times, exorbitant prices, and concerns about appropriateness for non-Western clients. Digital single-session interventions (SSIs) may offer a promising alternative. SSIs target particular mechanisms that underlie broad-ranging psychopathology, including deficits in problem-solving skills.

**Objective:**

Developed in the United States, Project SOLVE is a digital SSI that teaches problem-solving skills to adolescents. This study evaluated the acceptability, appropriateness, and utility of an adapted version, Project SOLVE-NZ, among *rangatahi* (young people) in Aotearoa New Zealand. Additionally, we evaluated a comparable online activity, Project Success-NZ, as a potential active control condition in a future randomized controlled trial of Project SOLVE-NZ.

**Methods:**

A sample of school students and teachers completed Project SOLVE-NZ and Project Success-NZ. Feedback on the interventions was collected through focus groups and semistructured interviews. Interviews were recorded, transcribed, and analyzed using reflexive thematic analysis.

**Results:**

In total, 12 students (aged between 13 and 14 years; female students: n=6, 50%) participated in a focus group, and 8 teachers (teaching experience: mean 8.75, SD 7.96 years; female teachers: n=5, 62.5%) participated in individual interviews. Participants endorsed the sociocultural relevance of Project SOLVE-NZ and Project Success-NZ to *rangatahi* in Aotearoa New Zealand and viewed all existing adaptations favorably. Participants felt that the interventions would be valuable to a wide range of *rangatahi*, helping to fill gaps in students’ learning and providing benefits to mental health. Participants also believed that the interventions may be particularly relevant for youths experiencing economic hardship. Interestingly, most participants had no preference for either Project SOLVE-NZ or Project Success-NZ, and they believed that both interventions could provide ongoing support to *rangatahi* throughout the school year. Teachers provided some suggestions on increasing student engagement with the interventions, namely, through increased cultural and gender representation, visual and literacy aids, *whakawhanaungatanga* (relationship building), and teacher guidance. Overall, interviews revealed that both interventions were perceived as acceptable, appropriate, and useful for *rangatahi* in New Zealand and highlighted further adaptations that could be made prior to a randomized controlled trial of Project SOLVE-NZ across schools nationwide.

**Conclusions:**

Digital SSIs show promise in addressing the mental health treatment gap for adolescents. Both Project SOLVE-NZ and Project Success-NZ were well-received by students and teachers in Aotearoa New Zealand and may provide benefits to youth mental health. We make the following recommendations for others interested in designing digital SSIs or similar tools for young people: involve *rangatahi* and relevant stakeholders in the design process, consider how the intervention will be implemented, ensure that the intervention accommodates a range of cognitive abilities, and ensure that the intervention reflects the diversity of *rangatahi* today.

## Introduction

### Background

Globally, we face a significant treatment gap in mental health care. Despite the existence of numerous evidence-based interventions, most individuals experiencing mental distress receive little or no formal support [1-3] for reasons including extensive wait times, exorbitant prices, and concerns about their cultural appropriateness for non-Western clients [4-8]. One alternative approach to existing treatment options may be single-session interventions (SSIs). Digital SSIs require less time and fewer resources than traditional psychotherapies and are increasingly—and effectively—being used in the treatment and prevention of various mental health conditions [9-15].

SSIs aim to address underlying, often transdiagnostic mechanisms or skill deficits implicated in broad-ranging psychopathology rather than provide comprehensive treatment for specific diagnoses. At present, SSIs have targeted numerous transdiagnostic mechanisms associated with psychopathology, including negative problem orientation [16], avoidance [12,17], cognitive distortions [9,10,13], and negative self-perceptions [18,19]. Problem-solving skills are another transdiagnostic mechanism linked to psychopathology [20-24] and targeted with digital SSIs [16,25]. In clinical psychology, problem-solving or “social problem-solving” is defined as the “self-directed cognitive-behavioral process by which an individual, couple, or group attempts to identify or discover effective solutions for specific problems encountered in their everyday living” [26].

In their seminal paper, D’Zurilla and Goldfried [27] theorized that individual differences in problem-solving ability are due to variance in two processes: (1) problem orientation and (2) problem-solving style. Problem orientation is the motivational force behind problem-solving, including the individual’s metacognitive beliefs about their problems and ability to solve them. Positive problem orientation views problems as solvable challenges, while negative problem orientation views problems as unsolvable and distressing threats. Problem-solving style refers to the idiosyncratic way an individual attempts to conceptualize their problems and generate effective solutions. Although some heterogeneity exists in stylistic approach, most individuals tend to be rational, impulsive or careless, or avoidant problem solvers. Rational problem-solving is the most adaptive problem-solving style and involves the deliberate and systematic application of problem-solving skills, including (1) defining the problem, (2) formulating multiple solutions, (3) deciding the “best” solution, (4) implementing that solution, and (5) monitoring the effectiveness of that solution.

It has been argued that some presentations of psychopathology are due to underlying problem-solving deficits, and improvements in these presentations require a shift to more positive problem orientations and rational problem-solving styles [26]. Indeed, this literature shows that deficient social problem-solving has been associated with a range of mental illnesses, including depression [20,24], anxiety [21,28], schizophrenia [22,29], and borderline personality disorder [23,30,31].

### Problem-Solving and Adolescents

Adolescents are a group who may particularly benefit from problem-solving interventions. Older adolescents (aged 13 to 14 years) show increased tendencies toward negative problem orientation, avoidant problem-solving style, and impulsive or careless problem-solving style compared to younger adolescents (aged 11 to 12 years) [32,33]. Additionally, marked sex differences have been observed, with rationality being more characteristic of girls in both age groups and impulsivity and avoidance being more characteristic of boys in the younger age group. Adolescence is also a developmental period marked by increases in stressors, which may benefit from problem-solving interventions, including interpersonal challenges, identity concerns, and worries about the future [34-36].

### Project SOLVE

Project SOLVE is a digital SSI developed by researchers at Harvard University [16]. Through a 30-minute self-guided intervention, Project SOLVE provides young people with adaptive strategies to face their everyday problems. Specifically, Project SOLVE aims to foster positive problem orientations and rational problem-solving styles among users: adolescents learn that some of their problems are solvable challenges using the “SOLVE” framework (ie, say what the problem is, one goal to aim for, list some solutions, vote for the best solution, and explore what works) rather than threats to be impulsively dealt with or avoided. A recent school-based randomized controlled trial (RCT) in the United States found that adolescent students who completed Project SOLVE showed significant reductions in internalizing symptoms at a 3-month follow-up assessment, relative to Project Success, an active comparison program focusing on study skills [16]. In addition, participating students reported significantly reduced hopelessness and increased goal-planning confidence from pre- to postintervention, and they rated Project SOLVE as acceptable and helpful.

### Project SOLVE and Aotearoa New Zealand

Like other developed nations, Aotearoa New Zealand is facing a growing mental health crisis, with adverse yearly trends in a variety of youth mental health indicators [37]. Converging systemic inequities contribute to poorer mental health outcomes for *rangatahi* (young people; a list of translated terms is provided in Multimedia Appendix 1) with 1 or more historically marginalized identities, including *Māori* youths (the indigenous people of New Zealand), Pasifika youths, and LGBTQIA+ (lesbian, gay, bisexual, transgender, queer, intersex, asexual, and other diverse sexual orientations, gender identities, and sex characteristics) youths [38,39]. One in five *rangatahi* report difficulty in accessing help for their mental and emotional concerns [38], and the New Zealand mental health care system has been “characterized by long delays, overworked staff, inadequate environments, and a lack of clear information” [7]. Additionally, there have been calls for the current system to be more responsive to the needs of *Māori* and Pasifika youths, who often have difficulty receiving effective and culturally appropriate care [8,40].

As a low-cost and low-resource digital SSI, Project SOLVE may be an effective means of expanding evidence-based treatment to *rangatahi* in Aotearoa. To this end, a school-based pilot study explored the perceived acceptability, helpfulness, and appropriateness of an adapted version of Project SOLVE for *rangatahi* in Aotearoa [25]. Adaptations included changes to the intervention vignettes, avatar, spelling, vocabulary, and overall aesthetic design and elicited overwhelmingly positive feedback from students using a brief qualitative survey (ie, the Program Feedback Scale [41]). However, it is important to note that these adaptations were guided by relevant literature and academic expertise rather than consultation with young people themselves. Indeed, consultation is an important aspect of cultural adaptation, as it provides individuals with the opportunity to “impact, lead, and shape the things that influence their lives” [42]. This process of consultation or “co-design” is particularly important in the context of Aotearoa, as current mental health services do not satisfactorily accommodate the diverse cultural needs, preferences, and world views of our people [7,8], and particular cultural groups (eg, *Māori* and Pasifika communities) have been historically excluded from and oppressed by Western research practices [43-46]. Ultimately, research methodologies that incorporate co-design allow mental health tools to be developed in collaboration with their intended recipients, which may improve their cultural appropriateness and overall efficacy.

### This Study

Building on the previous pilot study, we conducted a series of interviews with *rangatahi* and their teachers to gain a more in-depth understanding of their experience with the adapted Project SOLVE. Additionally, we asked participants about a comparable online activity, Project Success, that could be used as an active control condition in a future RCT of Project SOLVE. The interviews aimed (1) to determine the perceived acceptability, appropriateness, and utility of the SSIs for Aotearoa New Zealand and (2) to identify any further adaptations needed to the interventions prior to a nationwide RCT of Project SOLVE.

## Methods

### Ethical Considerations

All procedures across both the student focus group and the individual teacher interviews were approved by the University of Waikato Human Ethics Committee (HREC[Health]2024#15). Participants were provided with an information sheet about the study, and consent was attained prior to participation in an interview. They were informed that participation was voluntary, that they could decline to answer any question, and that they could leave the interview at any stage. Students and teachers received US $11.56 and US $23.12 vouchers, respectively, as *koha* (a gift) for their participation.

### Study Context

This study was conducted between October 2024 and March 2025. The student focus group was conducted in collaboration with a public high school in Aotearoa New Zealand with approval from the senior leadership team.

Methods were also guided by Sonja MacFarlane’s Cultural Enhancement Framework [47] (CEF)—a tool that has been used extensively by the New Zealand Ministry of Education to adapt programs originally designed for use overseas and aims to enhance the cultural appropriateness of *mahi* (work) done in New Zealand (Macfarlane, personal communication, June 2024). The CEF was particularly relevant for this study, as it provided a scaffolding to appropriately and ethically engage with participants, therefore increasing the likelihood of positive participant experiences and effective final products. For example, MTB met with leadership at the participating school to explain the purpose of this *mahi* and facilitate a *kōrero* (discussion) around any concerns or questions. We also worked with our school partners to ensure that parents were appropriately informed of the research, the (classroom) setting was conducive to *rangatahi* engagement, and *rangatahi* felt safe to express their *whakaaro* (thoughts and views) without fear or objection.

### Study Design

We conducted a qualitative study using focus groups and semistructured interviews. Focus groups involve a moderated discussion with a predetermined group of people to assess the opinions, beliefs, and experiences of a particular phenomenon [48]. Increasingly, focus groups, or similar style interviews, are being used by researchers to co-design mental health interventions with their intended recipients [49-51]. In this study, student feedback was elicited using focus group discussions, and teacher feedback was elicited using individual semistructured interviews following completion of Project SOLVE and Project Success.

### School Partnership

The majority of student participants were recruited from a large, public high school in New Zealand. This school is coeducational and categorized as “decile 1,” meaning that it is among the 10% of New Zealand schools with the highest proportion of students living in low socioeconomic communities. Additionally, the student population is disproportionately comprised of historically marginalized ethnic groups, with almost 60% and 30% of students identifying as *Māori* and Pasifika, respectively. (The most recent census reports 17.8% [887,493/4,993,923] of the New Zealand population as *Māori* and 8.9% [442,632/4,993,923] as Pasifika [52].)

In an effort to align with culturally sensitive practices, we consulted the CEF as well as the participating schools’ deputy principal and head of physical education and health on how to best work with this specific community (eg, methods of gaining parental consent, determining participant compensation, and building positive rapport with students).

### Student Participants

From the school partnership, 2 classes of year 9 students (ages 13-14 years) were invited to participate in this study. All students in these classes completed the interventions during a regularly scheduled health lesson. All students were also invited to participate in a focus group interview outside of class hours, which required parental consent.

### Teacher Participants

First, health and physical education teachers from the partner school were invited to participate in this study. Based on these 2 interviews, we then used snowball sampling to recruit additional teachers from schools of other socioeconomic and cultural contexts to gain alternative perspectives on the interventions. Teachers gave informed consent before participating.

### Interventions

#### Project SOLVE-NZ

Project SOLVE is a 30-minute self-guided activity delivered via Qualtrics, an online survey platform. First, students are shown a welcome screen that introduces the activity and the avatar, *Māia*. *Māia* then guides the students through a series of modules, including an introduction to problem-solving and which types of problems might be most appropriate for this skill, a description of how the brain facilitates problem-solving, vignettes demonstrating how older adolescents have solved their problems, scientific evidence that problem-solving can work, practice exercises, and activities to encourage the use of problem-solving in daily life. The intervention teaches students how to solve problems via the “SOLVE” framework: say what the problem is, one goal to aim for, list some solutions, vote for the best solution, and explore what works. In this study, participants completed Project SOLVE-NZ, the adapted version of Project SOLVE used in the previous pilot study in New Zealand [25]. This version included voiceover recordings of the paragraph text to accommodate diverse learning needs.

#### Project Success-NZ

Project Success is comparable to Project SOLVE in format and length and was used as an active controlled condition in a previous RCT of Project SOLVE [16]. Instead of problem-solving skills, Project Success teaches young people 3 strategies to reach their academic goals: how to take effective notes, how to break big assignments down into smaller tasks, and how to ask trusted others for help. Like Project SOLVE, Project Success was originally intended for use among American youths. Therefore, adaptations were made by MTB prior to this study to create Project Success-NZ (ie, using voiceover recordings, the same aesthetic design, avatar, New Zealand English, *te reo Māori* [the *Māori* language], and vocabulary more common to *rangatahi* in New Zealand). These adaptations matched Project Success-NZ to Project SOLVE-NZ to ensure that any future RCT comparisons between the interventions were based on content rather than superficial differences.

### Procedure

#### Students

On the study day, students attended their usual health class, where MTB welcomed them and engaged in *whakawhanaungatanga* (relationship building), sharing brief information about herself and the study. Using laptops, students were emailed a Qualtrics link to read the study information and assent form. MTB also explained the process orally to support diverse learning needs. Students then completed demographic questions (age, gender, and ethnicity) before working through Project SOLVE-NZ (problem-solving skills) and Project Success-NZ (academic strategies) at their own pace.

Two 30-minute focus groups were held later that day during morning interval and lunch, led by MTB using a semistructured guide. MTB, a first-year postgraduate student in clinical psychology at the University of Waikato, had no prior relationship with the students. *Karakia* (prayer) opened and closed each session and *kai* (food) was provided.

#### Teachers

Teachers received a Qualtrics link to the interventions via email and could complete them at their convenience before their interview. MTB conducted individual ~20-minute semistructured interviews with each teacher via Zoom (Zoom Video Communications) or phone, based on their preference. *Karakia* was also offered.

### Analysis

Focus group discussions and individual interviews were audio-recorded and transcribed with assistance from Otter.ai (version 3.8.0; Otter.ai, Inc). MTB then anonymized the transcripts to preserve participant confidentiality. After familiarizing herself with the transcripts, MTB coded the data using the “comments” function in Microsoft Word (version Microsoft 365; Microsoft Corporation). These codes were then distilled and organized visually into themes using Miro, a digital workspace for collaborative brainstorming. Themes were reviewed by coauthors and supervisors and consolidated through discussions with MTB. The transcripts were revisited throughout the analysis to ensure that the findings remained grounded in the original data. To illustrate the themes, participant quotations are presented in the subsequent sections, annotated with participant number, self-identified gender, and age (if a student) or years of experience (if a teacher).

## Results

### Participant Characteristics

#### Students

A total of 12 students aged between 13 and 14 years participated in a focus group (mean 13.67, SD 0.49 years), with an even number of male (6/12, 50%) and female (6/12, 50%) students. Half of the participants identified with only 1 ethnicity, while half identified with multiple ethnicities. Pasifika ethnicities were most represented (Samoan: n=6; Tongan: n=3; Cook Islands *Māori*: n=2; Fijian Indian: n=1), followed by *Māori* (n=4), New Zealand European (n=4), American (n=1), and Indian (n=1).

#### Teachers

A total of 8 teachers participated in individual interviews, with 5 identifying as female teachers. The teachers ranged in teaching experience from 1 to 23 (mean 8.75, SD 7.96) years. In total, 6 teachers identified as New Zealand European, with the remaining 2 identifying as “other.” The teachers represented 7 schools of varying socioeconomic and cultural backgrounds, including single sex, coeducational, low decile, middle decile, and high decile ranking.

### Thematic Findings

#### Major Themes

Participant feedback was categorized into 4 themes, with each theme containing multiple subthemes. These subthemes were labeled as either positive (+), negative (–), or mixed (+ or –), as shown visually in Table 1.

**Table 1:** Qualitative feedback of rangatahi and teachers about Project SOLVE-NZ and Project Success-NZ organized into themes and subthemes.

Theme	Subtheme
Sociocultural relevance	Avatar of Māia (+) Vignettes (+) Te reo Māori (+) Relevance to youths experiencing socioeconomic inequities (+)
Value of interventions	Learning new skills (+) Benefits to mental health (+) Past, present, and future value (+) Pedagogy of interventions (+) Difficulty remembering content (–)
Delivery of interventions	Digital delivery feasible (+) Class setting appropriate (+) Self-guided may present challenges (+ or –) Voiceovers (+)
Suggestions	Increased diversity Visual and literacy aids Whakawhanaungatanga Teacher support

#### Theme 1: Sociocultural Relevance for Aotearoa New Zealand

##### Overview

Students and teachers unanimously agreed that Project SOLVE-NZ and Project Success-NZ were appropriate for use in Aotearoa New Zealand. Specifically, the majority of students described *Māia* (the avatar) as “cool” and “relatable” (11/12), with 1 male student disagreeing. These feelings were reflected in the comments of teachers, who liked the design of *Māia* but thought a female character may limit the engagement of young boys:

I don’t know whether you would want to make it a bit more ambiguous about their gender, or whether you just make it male ... If you had a boy’s version of that image, that might be more relatable for them.

Teacher 6, female, 2 years of experience

I think with some boys, especially that age, it’s really hard to get them to be honest with themselves, and a female figure could just be another barrier to them opening up.

Teacher 7, female, 16 years of experience

Participants also provided positive feedback on the adapted vignettes. All students reported liking the vignettes, saying that the selection included figures that students could “idolise” (Student 3, female) and “really connect to” (Student 1, female) and provided “good examples [of problem solving] because they were all from New Zealand” (Student 2, female). Overall, teachers also had positive experiences with the vignettes, commenting that they were “fabulous choices” (Teacher 4, female) and “relatable to a New Zealand audience” (Teacher 8, male). One teacher was unsure whether young people would recognize Valerie Adams (a New Zealand Olympian who retired in 2022), but all students reported knowing who she was.

Finally, participants spoke to the inclusion of *te reo Māori*, which was appreciated by the students and believed to facilitate their learning: “We grew up learning te reo and [including] it will help us understand more” (Student 2, female).

Furthermore, the students did not believe that including *te reo Māori* would be a barrier to nonspeakers, citing the English translation and commenting that young people “should learn te reo if [they] are growing up in New Zealand” (Student 2, female). Incorporating *te reo Māori* means that nonspeakers “can get to know our [Māori] culture better” (Student 3, female).

Overwhelmingly, this perspective was shared by the teachers; however, one commented that including additional cultures could better represent the diversity of classrooms across Aotearoa:

It was great that it had te reo in it ... I just think, for New Zealand, it could be in different languages every now and then. Like Māori and then maybe Chinese. Just to better reflect our makeup now.

Teacher 5, female, 10 years of experience

##### Relevance to Youths Experiencing Socioeconomic Inequities—“Those kids end up missing out ...”

Discussions on the general sociocultural relevance of Project SOLVE-NZ and Project Success-NZ also brought to light the specific relevance to youths experiencing socioeconomic inequities. One teacher noted how the economic disadvantage of many families in Aotearoa seems to affect the development of problem-solving and study skills in their children:

These skills are generally transferred to children through their parents, but a lot of our kids don’t necessarily live with their parents, or they live with other caregivers, or there’s a lot of other kids in the house. The parents are just so busy trying to earn a living that they don’t have the time to go through those skills or the patience to teach those skills, so those kids end up missing out ...

Teacher 7, female, 16 years of experience

While this perspective implies that both interventions could be particularly valuable to youths facing socioeconomic hardship, another teacher commented that Project Success-NZ may be less relevant to students from low-decile schools, as they tend not to prioritize academic achievement:

I would say very few of our students study. Some might, but the general culture within the school would be no. I mean, they don’t even do homework ...

Teacher 1, male, 23 years of experience

However, he continued by acknowledging the intrinsic value of study skills and expressed a desire for these students to still have that learning opportunity:

But that doesn’t mean these students shouldn’t understand this is actually how you do it. If you want to study, this is what you can do—this is what you have to be able to do to achieve academically.

Teacher 1, male, 23 years of experience

Another teacher agreed on the general importance of learning study skills but ultimately believed that Project SOLVE-NZ may have more relevance in low socioeconomic contexts:

I thought the content was really beneficial ... But I think the problem solving one is probably a good one to focus on at this [low socioeconomic] school. Not saying that the academic stuff isn’t important, but it would probably be helpful to figure out what the target audience is [of the interventions] and how relevant study skills actually are.

Teacher 2, female, 1 year of experience

However, this sentiment was not shared by the students at this school, the majority of whom had no preference for either intervention (9/12).

#### Theme 2: Value of Interventions to Students

##### Overview

In addition to sociocultural relevance, students and teachers commented on the value of Project SOLVE-NZ and Project Success-NZ to *rangatahi* in New Zealand. Specifically, participants described the interventions as tools to learn new skills and improve mental health through ongoing engagement and appropriate pedagogy.

##### Learning New Skills

Many teachers believed that the interventions would provide an opportunity for students to learn skills they may not be familiar with. In terms of Project SOLVE-NZ, teachers described the intervention as an effective way for students to learn a more adaptive approach to overcoming their daily challenges:

[Students] actually don’t have the skills to say: “Okay, this has happened. Let me figure it out. Am I going to try and make new friends in the netball team? Am I going to go to the chess club because I love chess? Am I going to ask mum if I can have some new classmates come over for a sleepover?” They need to be stepped through those suggestions or problem-solving strategies because it’s not embedded in them yet.

Teacher 7, female, 16 years of experience

These perspectives were also reflected in the student comments, with participants sharing that Project SOLVE-NZ taught them how to “take on overwhelming situations” (Student 1, female), “manage [their] problems” (Student 12, male), “break down [their] problems” (Student 5, female), and “find the best way to solve a problem” (Student 6, female). One student also noted the link between learning problem-solving skills and emotion regulation:

[We] didn’t learn how to problem solve ... It hasn’t been explained to us in a good way, so then when it comes to problem solving, we just get angry.

Student 2, female

According to participants, Project Success-NZ also provided *rangatahi* with an opportunity to learn valuable skills. Indeed, teachers unanimously agreed that study skills are important for students to know, and some questioned whether presuming competency in skills like those taught in Project Success-NZ is justified:

One of the things that dawned on me is, like, I don’t actually know if students have ever been taught to take notes before—how to do that effectively. So I think, as a crash course, [Project Success–NZ] is pretty cool. It made me reflect on my own practice and whether I need to spend more time teaching those skills. Because I’ll put up slides or do a talk, and part of me just expects that students are going to take note of a few things I say, but they really just sort of look at me ...

Teacher 8, male, 4 years of experience

Additionally, teachers believed that Project Success-NZ would provide a beneficial learning opportunity for students across academic abilities:

I think whether they’re high achieving students that are being extended and have tons of pressure on them, or they’re lower achieving and struggling just to get an "Achieved" [grade], [Project Success–NZ] is going to help them figure out what study skills are going to work for them.

Teacher 6, female, 3 years of experience

##### Benefits to Mental Health—“I feel like SOLVE would really help a lot of kids”

Students perceived that Project SOLVE-NZ could have a positive impact on *rangatahi* mental health. For example, one student commented that Project SOLVE-NZ could help students “get to know their body, thoughts, and feelings better” (Student 5, female), while another commented explicitly on the mental health crisis facing *rangatahi* in Aotearoa today:

I feel like SOLVE would really help a lot of kids ... Because New Zealand has a very high, like, mental health rate, I feel like if [Project SOLVE–NZ] were given out to kids across New Zealand, it would really help them.

Student 1, female

Teachers agreed that Project SOLVE-NZ could provide benefits to youth mental health and identified some potential benefits of Project Success-NZ. For instance, one teacher thought that Project SOLVE-NZ may help students “move toward their purpose and develop forward thinking” (Teacher 1, male, 23 years of experience). He then expanded by linking Project SOLVE-NZ to self-autonomy:

I think if students can learn that some things are in their control, that’s everything. You’re more getting taught now that the tough things happening to you are not really because of you and you don’t have control of this or that ... But I think that you can still achieve, you can still do this. And I think going back to that message is good.

Teacher 1, male, 23 years of experience

This teacher also noted how problem-solving skills may help students cope with the adversity in their lives:

A lot of our students are in survival mode, which means they’re only really thinking an hour ahead ... So I find the forward thinking is not quite there. It’s almost like “When life becomes easy, then we can start thinking about the future.” But it’s not like that. Life is hard, so they have to learn these skills.

Teacher 1, male, 23 years of experience

A similar sentiment was shared by this teacher, who believed that Project SOLVE-NZ may provide benefits to mental health by bridging the gap between students’ aspirations and reality:

They need things to help them cope with [their problems] because even though we’ve got TikTok and Snapchat and all the different apps that kids use, there’s nothing in those apps that give them the tools. They just see what they have to be like, or what they need to be like. They don’t have that middle block of, “How do I get there?” Or, “How can I be my own self and get to express myself in that way?” They don’t have those tools currently ...

Teacher 7, female, 16 years of experience

##### Past, Present, and Future Value—“I wish I knew this last year before I got suspended”

Both students and teachers commented on the value of the interventions at earlier and later stages of development. All students agreed that ongoing access to Project SOLVE-NZ would be helpful, and one student noted how younger age groups could benefit from problem-solving skills too: “This would have been really helpful in intermediate because everyone has emotions and problems—even when we are younger” (Student 1, female).

A similar sentiment was shared by this student, as she reflected on her younger self: “I wish I knew this last year before I got suspended” (Student 2, female).

Teachers agreed that students are beginning high school with underdeveloped problem-solving skills, with one thinking that this trend is getting worse over time:

With the kids we see coming into high school now, a lot of them lack problem solving skills and the ability to actually figure out how to study and write notes correctly. And I think it’s deteriorating.

Teacher 3, male, 1 year of experience

He also believed that earlier exposure to Project SOLVE-NZ could be helpful:

I think it’s even good for kids in a younger setting to have a look at it early on, so that when they go to high school, they already have some familiarity with the skills.

Teacher 3, male, 1 year of experience

Teachers, in general, endorsed ongoing use of the interventions throughout the school year. For example:

I would say it needs to be an ongoing program. [Project] SOLVE is fabulous, but what’s next? Are they reminded to use it again? What’s the prompt for them to use it each time they have a problem? It needs to be in your face. Like it needs to be something that’s not just going to disappear. Something that’s a bit regular and talked about.

Teacher 4, female, 12 years of experience

##### Appropriateness of Pedagogy—“I thought it was a neat approach”

For the most part, students and teachers had positive experiences with how the interventions taught problem-solving and study skills. Specific strengths included the overall layout, which teachers considered would be effective and engaging for their students:

I thought it was really well done, honestly. I thought it was a neat approach to something that’s not necessarily that easy to talk about without either being boring or repetitive and saying the same thing. I really like that. It gives them options to choose from, and they have to think about what they’re doing.

Teacher 6, female, 3 years of experience

Participants also liked the use of multiple-choice and short-answer questions to break down the content in a digestible way:

I liked how there was a mix of like multi choice and short written response questions. Because I think that the shorter the response, the better it is for the younger kids. And even though they had to think for the answers, they didn’t have to write a paragraph explaining it—they could just fire down whatever was in the top of their mind ... Otherwise they’re like “Oh my God, how much do I have to write?”

Teacher 5, female, 10 years of experience

Students enjoyed practicing the skills by helping fictitious young people with their problems: “I liked how they put us in the shoes of the characters, and we could answer the questions from their perspective” (Student 1, female).

Teachers liked the selection of exemplar problems, commenting on how students could cater the intervention to their specific interests or needs:

It was kind of like that Bear Grylls show where you can choose the ending. It gives them some agency, which I thought was cool, because they can see how the decisions that they make are going to impact the way that they solve a problem. And it gets them thinking, which is always good.

Teacher 6, female, 3 years of experience

Finally, one student liked the privacy of a tool that is online and self-guided: “There’s more privacy. You’re able to open up and share what you’re going through” (Student 6, female).

The only negative feedback regarded difficulty remembering content from the interventions. For example, students retained the general underpinnings of Project SOLVE but struggled to recall what each part of the acronym stood for. This difficulty was foreseen by teachers:

The content is really good, and I definitely agree that it’s super important to get out to them. It’s just, how do you get them to retain it and not just have it fly out of their mind.

Teacher 2, female, 1 year of experience

They’re probably not going to remember 80% of what goes on in the middle of the activity, but if they can take away the acronym—a step-by-step breakdown about how they could solve their problems—then they could reflect on it and use it when those issues and conflicts present themselves in their day to day life.

Teacher 8, male, 4 years of experience

#### Theme 3: Delivery of Interventions in Schools

##### Feasibility of Digital Delivery—“Most of our lessons are supposed to be designed around technology now”

The majority of students preferred doing activities online (11/12), and teachers said that most schoolwork is now delivered digitally. One teacher highlighted how young people’s familiarity with and preference for digital content needs to be acknowledged when encouraging help-seeking behavior:

Yes, [students] need tools and things to deal with people and have social relationships in person, but a lot of who they are is online ... I think something like [these interventions] are a really good “can opener” for those kids. They need to find something to open themself up to what they’re really feeling. How could they cope with it? What could they do?

Teacher 7, female, 16 years of experience

More practically, most teachers had few concerns about students’ access to digital devices to complete the interventions:

There would be no issues with [access to devices] at our school. There’s so much funding for devices now, and so much of the teaching we do needs a device. So I would say nearly all schools would have access to them.

Teacher 3, male, 1 year of experience

However, one teacher recounted her difficulty with online activities and cautioned that some schools may have limited internet access:

At [my current school], we’re now fully BYOD, so every kid has a device ... But not all schools have internet access. At [my previous school], I was in a classroom and wanted to watch a video, but the students said, “No, you can’t do that in this classroom. The internet is too bad.”

Teacher 5, female, 10 years of experience

##### Appropriateness of Classroom Setting—“They say they’ll do it at home, but they won’t”

The majority of students (10/12) preferred to complete the activities at school, rather than at home. These students felt a class setting would be more conducive to their engagement and understanding:

I feel like you’re also more likely to do it properly and then remember it later on in the day if you get too overwhelmed. If you’re just like, “Oh yeah, that thing I learned a couple hours ago, I can use it here.”

Student 1, female

Students also noted how help is more easily accessible in a class setting, and there tend to be less distractions in class than at home:

There’re no distractions at school, and it was helpful to have other people around me.

Student 6, female

If I needed help, I could just look over at my friends’ work, and, at school, you don’t have dramas with anybody ... you don’t have people coming in and out of your house all of the time distracting you.

Student 2, female

In contrast, 2 students felt as though their engagement with the interventions would be better at home: “There is more discipline at home, so I would be able to concentrate on it better” (Student 4, female).

All teachers believed that it would be better to complete the interventions in class, noting that some students would not appropriately engage with the interventions outside of school hours, if at all:

They won’t [do it at home]. They say they’ll do it at home, but they won’t. Maybe one might. One or two, and it’ll be as a very specific type of kid.

Teacher 1, male, 23 years of experience

The majority of students (10/12) agreed that they were unlikely to do it at home without teacher instruction (“I’m just not gonna do it at home” [Student 11, male]) or because their home lives are busy with other priorities (“I’ve got chores to do [instead]” [Student 5, female]).

##### The Challenges of Self-Guided Interventions

The self-guided nature of the intervention received mixed reviews. Students noted how they felt their class was more settled and engaged than usual when completing the interventions: “It was really quiet, and there was nothing flying around” (Student 2, female).

Additionally, most teachers thought that self-guided activities would be appropriate for their students. For example:

I don’t think it would be an issue for our boys to be led by themselves. I think they’re used to doing that, so I don’t see that as being a problem ... There might be some students that struggle to get started, but you would just go and work with them one-on-one.

Teacher 6, female, 3 years of experience

I’m a big fan of group work ... but I think when you’re trying to get strong outcomes, it’s quite difficult to get that in a group setting, and so I think getting kids independently to work through it with headphones would be a really good way of doing that.

Teacher 8, male, 4 years of experience

However, teachers were also conscious that the success of self-guided interventions may vary between schools and within classroom settings:

Sometimes you’ll have a super academic class who would absolutely thrive in those surveys, and sometimes you’ll have people who can’t sit still for 10 minutes in the class.

Teacher 2, female, 1 year of experience

##### The Role of Voiceovers—“It helped us understand more”

All students reported that the voiceovers were helpful. For example:

[The voiceovers] were actually really helpful because sometimes I just can’t be bothered reading, so I multitask, and I listen to it and do the work at the same time.

Student 2, female

Another student explained that the voiceovers helped her understand the content better:

I feel like if we were to read it, we would just skim through it ... We would not really take it in. The voiceover was slow, and it helped us understand it more.

Student 1, female

Teachers also endorsed the inclusion of voiceovers, noting the low literacy of many *rangatahi* in New Zealand and how this can impact learning:

Literacy is terrible ... so I think listening to it is definitely a lot better. Some kids will read it and be able to read it fast as, but a lot of kids, especially if it’s aimed at Year 9s and Year 10s, would definitely want to listen to it.

Teacher 3, male, 1 year of experience

I think you definitely would have engaged them more by having someone talk to them, rather than them just sitting there trying to read the same thing 100 times because it doesn’t make sense.

Teacher 2, female, 1 year of experience

Experiencing an increase in neurodivergence among her students, one teacher also felt that the voiceovers would be crucial to accommodate learning differences:

There’s a massive amount of dyslexia coming out now as well. [The voiceover] just helps take away that struggle that can hinder them from actually trying, because “Oh, I’ve gotta read all this stuff,” or “I don’t know how to read it, so I don’t know how to respond.”

Teacher 7, female, 16 years of experience

#### Theme 4: Suggestions for Improvement

##### Overview

Participants were asked how the interventions could be improved. While students did not suggest any improvements, teachers believed that getting students to “take [the interventions] seriously” would be the biggest challenge:

I think the only challenge will be how can we make sure that everyone’s going to take it seriously? Because obviously some people will really like it—that’s the same with everything—but how do we make sure everyone gets the most out of it they can?

Teacher 3, male, 1 year of experience

These concerns in mind, teachers suggested improving student engagement through (1) increasing the cultural and gender representation throughout the interventions, (2) adding more visual and literacy aids to supplement paragraph text, (3) engaging in *whakawhanaungatanga* with students, and (4) providing adequate support to teachers so they can confidently implement the interventions.

##### Increasing Diversity—“To better reflect our makeup”

Teachers felt that the interventions could better represent the diversity of classrooms in Aotearoa. As mentioned previously, teachers believed that having a boy character to accompany *Māia* may increase the engagement of male students. One teacher also noted room for increased cultural representation in the interventions.

##### Adding More Visual and Literacy Aids—“A range of learners”

As mentioned previously, teachers liked the content of the interventions but felt that students may struggle to remember the skills. Teachers suggested that more visuals or animation throughout the interventions might help students better grasp the concepts:

I find that having more visuals for them to latch on to probably would help them remember a bit better, particularly with the range of learners that might be neurodiverse and can’t sort of engage so much with the text version, they might be able to engage more with the visual aspect of it.

Teacher 8, male, 4 years of experience

Additionally, one teacher also noted that a shorter video in Project Success-NZ could be more effective: “The only thing was that I did find the video a bit boring, but that might just be my ADHD brain ...” (Teacher 4, female, 12 years of experience).

Students also discussed ways to remember the skills, with most students liking the idea of a follow-up email reminder or a poster displayed in their classroom. One student took a photo of the SOLVE skill so she could remember it; others said it would have been helpful if their teacher prompted them to do so. No student thought that a handout would be helpful, with most believing “a paper [reminder] is just going to get lost” (Student 1, female).

In terms of literacy concerns, teachers praised the inclusion of voiceovers, but one suggested further addressing literacy challenges by adding definitions:

Literacy and reading are terrible, so even if you think there’s a word that might be a little bit complicated for them to understand, you could always have a little definition at the bottom as well.

Teacher 3, male, 23 years of experience

Another teacher believed that it would be helpful for the word to be highlighted as the voiceover is playing:

Some students have got such low literacy, so the voiceovers were fantastic. And as the voiceover is voicing, it could even be good to highlight each word. That would be very helpful for students too, especially ESOL students.

Teacher 4, female, 12 years of experience

##### Engaging in Whakawhanaungatanga—“Relationships are the most important thing”

Many teachers felt that student engagement would rely on the strength of the relationship between the class and the teacher delivering the intervention: “I think if the teacher has some good rapport or a good relationship with the learners they will buy into it more” (Teacher 8, male, 4 years of experience).

Another teacher commented how relationship building or *whakawhanaungatanga* is especially important at schools with high Māori and Pasifika populations “because relationships are the most important thing at a school like this to ensure [the students] actually care” (Teacher 2, female, 1 year of experience).

##### Providing Teacher Support—“The teacher knows the students best”

As future champions of these interventions, teachers felt that a support resource could be helpful in increasing student engagement. One teacher noted:

I think engagement comes down to the attitudes of the students a little bit, which you have to push as a team—as teachers. But if the teachers are educated—if there’s some teaching before it and the boys understand, or the students understand what the point of it is—then it might work better.

Teacher 3, male, 1 year of experience

Another teacher commented how it might be helpful for teachers to know more about the interventions and what they are (and are not) designed to target:

Maybe you could develop [that support piece] and be like, “Well, you know, this is the realm of sort of ‘normal problems.’ But some people might be experiencing things outside of this, and if that’s the case for you, that’s okay. Here’s some other things that you can do.”

Teacher 6, female, 3 years of experience

Teachers also felt that there would need to be flexibility in delivery, providing teachers with delivery options so they can cater to the specific needs of their class or individual learners:

I don’t think you could just give them something and say, “This is how you’re going to teach it.” I think there would need to be options and for teachers to be able to put their little twist on it because, at the end of the day, that is the teacher that has built the relationship and knows those students the best.

Teacher 2, female, 1 year of experience

For example, one teacher felt as though small groups, rather than self-guided, would work best for his class:

I find that one of the secrets to engagement is getting them talking about it. If you can get them talking, you’re kind of winning ... I feel that could be done with your activity. I think the ideal thing would be the students in small groups, and they’d be discussing it.

Teacher 1, male, 23 years of experience

Although beneficial in general, it was also noted that the helpfulness of a support resource may be dependent on the experience of the teacher:

Maybe for the first-year teachers, [a support resource] would be really beneficial ... but with more teaching, you’d know what you’re wanting out of the kids, and I would feel confident just doing [the activities] off the cuff.

Teacher 5, female, 10 years of experience

## Discussion

### Principal Findings

This study explored the experience of students and teachers with 2 digital SSIs: Project SOLVE-NZ and Project Success-NZ. Participants reported that the adapted interventions felt relevant for *rangatahi* in Aotearoa New Zealand, liking the character of *Māia*, the selection of vignettes, and the inclusion of *te reo Māori*. Participants also felt that both interventions would be valuable for a broad range of students by building their skills and supporting their mental health and may be especially helpful for youths experiencing socioeconomic hardship. Students and teachers agreed that the interventions would be best delivered in class, with fewer distractions and greater access to technology than most homes or other settings. Teachers provided some suggestions on increasing student engagement with the interventions, namely, through increased cultural and gender representation, visual and literacy aids, *whakawhanaungatanga*, and teacher guidance. Overall, participants viewed both interventions as acceptable, appropriate, and useful for *rangatahi* in Aotearoa New Zealand while identifying further adaptations to consider before an RCT of Project SOLVE-NZ nationwide.

### Comparison With Prior Work

At present, the body of literature on digital SSIs is emerging and primarily focuses on their potential utility as mental health support for young people. While the results of these studies have been favorable, the bulk of research has been conducted with American youths, making it unclear whether digital SSIs have utility in other parts of the world. This study sought to adapt existing digital SSIs for use among *rangatahi* in Aotearoa, using feedback from students and teachers. A similar study was conducted by Wasil et al [51] with students and teachers in India; however, these digital SSIs focused on gratitude, growth mindsets, and behavioral activation. Interestingly, the Indian students and teachers commented on different aspects of their digital SSIs to the New Zealand participants in this study. For instance, Indian teachers largely focused on how the content of the SSIs could be improved (eg, including neuroscience concepts), while our New Zealand teachers were more concerned about optimizing the interventions’ delivery (eg, ensuring that teachers had enough flexibility to accommodate their learners). Furthermore, the Indian students were seemingly forthcoming with ways to improve their digital SSIs (eg, how to make the vignettes more relatable), whereas the New Zealand students made no suggestions for improvement and claimed to be accepting of the interventions as is. On the one hand, this finding may suggest that Project SOLVE-NZ and Project Success-NZ are particularly well suited to the New Zealand context. On the other hand, it is possible that the *rangatahi* in this study did not feel comfortable voicing their suggestions for or criticisms of the interventions. Indeed, the majority of students in this study identified as Māori or Pasifika, and these communities have been historically harmed by Western psychological research practices and individuals of European descent (of which MTB is) [43-46]. Therefore, it is possible that the *rangatahi* in this study felt subject to a relational power dynamic in which criticism would be unacceptable.

### Recommendations

Based on our participants’ experiences, below are recommendations for researchers and developers of digital interventions for adolescent mental health.

#### Involve Rangatahi and Relevant Stakeholders in the Design Process

Researchers should work directly with *rangatahi* to ensure that the intervention meets their needs, as the youths in our study identified several areas for further adaptation that had not been considered by academics or teachers. In addition, engaging relevant stakeholders—such as teachers, parents, and coaches—can provide useful collateral information about the needs of *rangatahi* and potential challenges related to implementation.

#### Consider How the Intervention Will Be Implemented

Implementation success is likely to vary depending on factors such as the suitability of the environment in which the intervention is delivered (eg, school vs home), the relationship between the implementer and the *rangatahi* (such as researcher-*rangatahi* vs teacher-*rangatahi*), and the design of the intervention itself (for instance, a single session vs repeated engagement).

#### Ensure the Intervention Accommodates a Range of Cognitive Abilities

This may include providing a voiceover option, defining complex words, supplementing text with photos or videos, keeping the intervention to approximately 20-30 minutes where possible, and following up with brief content reminders, such as sending *rangatahi* a graphic summarizing what they learned.

#### Ensure the Intervention Reflects the Diversity of Rangatahi Today

If characters are used, offering options that represent a range of gender identities and cultural backgrounds is beneficial. In addition, developers should aim to balance representations of Indigenous cultures with those of other historically marginalized communities or cultural minorities.

### Strengths and Limitations

This study provided in-depth qualitative data on the experiences of *rangatahi* and teachers with Project SOLVE and Project Success. In analyzing participant responses, we were able (1) to deduce the acceptability, appropriateness, and utility of Project SOLVE-NZ as a digital SSI for adolescent mental health in Aotearoa New Zealand and (2) to establish whether Project Success-NZ would be a comparable active control condition in a future RCT of Project SOLVE-NZ nationwide.

Nonetheless, this study has some notable limitations. The sample of participants was gender balanced; however, all students were sampled from one low-socioeconomic school in New Zealand. Consequently, the sample of students consisted mostly of Māori and Pasifika young people, who are presumed to come from low socioeconomic backgrounds. This school partnership was purposeful; we wished to co-design Project SOLVE-NZ and Project Success-NZ with the *rangatahi* who likely have the most therapeutic need and potential benefits from a low-cost and low-resource intervention. Indeed, some of the students believed that the interventions would be particularly helpful for Māori and Pasifika youths; however, they also endorsed the use of the interventions across schools in New Zealand. The teachers in this study shared a similar sentiment, with some highlighting the applicability of the interventions to Māori and Pasifika youths, specifically, and others citing the potential benefits of the interventions to youths regardless of their cultural identity or socioeconomic status. That said, future research is needed to confirm how other young New Zealand people—of wide-ranging ethnic and socioeconomic identities—experience these interventions.

It is also possible that participants demonstrated social desirability bias in the interviews, concealing their criticisms about the interventions to please the interviewer. Attempts to mitigate this bias were made, with MTB having no prior connection to the students and encouraging honesty throughout the focus groups. However, as mentioned previously, no student made a suggestion on how the interventions could be improved and may have felt voicing criticism was unacceptable. Students may have also had limited insights into improving the interventions, as problem-solving and study skills appeared to be relatively new concepts. MTB knew 2 of the teachers whom she interviewed, but it was noted that these teachers provided constructive feedback and suggestions on improving the interventions going forward. Future research could use anonymous self-report questionnaires to gather additional data on students’ experiences.

MTB checked that all students completed the interventions before their focus group interview; however, there was no assessment of whether the interventions were completed appropriately (eg, checking the quality of a student’s response to the intervention questions). Therefore, it is possible that some students participated in the focus group without engaging with the interventions as the researchers intended. Looking ahead, it will be crucial to examine the quality of engagement with Project SOLVE-NZ and Project Success-NZ in order to obtain a valid indication of their effects on *rangatahi* mental health.

Finally, there is an opportunity for greater involvement of *rangatahi* in the development of these interventions and others similar. While this research integrated elements of co-design (ie, attaining user feedback to guide future adaptations), “true” co-design would develop a problem-solving intervention with *rangatahi* from the ground up. Indeed, earlier involvement of *rangatahi* may better guide the framing and pedagogy of an intervention, which could lead to greater skill development and mental health outcomes. True co-design with diverse *rangatahi* may also highlight needs and considerations that are specific to certain groups (eg, accessibility concerns for students with learning challenges).

### Conclusions

Digital SSIs are a promising means of closing the treatment gap in mental health care. Our findings suggest that Project SOLVE-NZ, an adapted 30-minute digital SSI that teaches problem-solving skills, is acceptable and appropriate for youths in Aotearoa New Zealand and was perceived to have potential benefits to youth mental health. Additionally, we found evidence that Project Success-NZ, a comparable digital SSI that teaches study skills, was also well received. Overwhelmingly, *rangatahi* and their teachers endorsed the use of Project SOLVE-NZ and Project Success-NZ in high schools across New Zealand, citing perceived benefits to adolescents from diverse cultural and socioeconomic identities. Teachers also provided suggestions on how to enhance student engagement with the interventions in the future.

Beyond Project SOLVE-NZ and Project Success-NZ, our findings suggest that digital tools for youth mental health may benefit from involving *rangatahi* and relevant stakeholders in the design process. This involvement could support researchers to build interventions that appeal to *rangatahi*, accommodate a range of cognitive abilities, represent diverse identities, and overcome the challenges of implementation [42,53-55]. Informed by this study, we look forward to examining the effects of Project SOLVE-NZ and Project Success-NZ on *rangatahi* mental health outcomes in the future.

## Appendix

Multimedia Appendix 1 Translation of te reo Māori words.

## Abbreviations

BOYD: Bring your own device
CEF: Cultural Enhancement Framework
LGBTQIA+: lesbian, gay, bisexual, transgender, queer, intersex, asexual, and other diverse sexual orientations, gender identities, and sex characteristics
RCT: randomized controlled trial
SSI: single-session intervention
