# Supplementary material for: Exploring the Acceptability, Appropriateness, and Utility of a Digital Single-Session Intervention (Project SOLVE-NZ) for Adolescent Mental Health in New Zealand: Interview Study Among Students and Teachers
Source: JMIR Form Res. 2026 Jan 13;10:e81259. doi: 10.2196/81259 (PMC12848488; doi:10.2196/81259)
Supplement: Multimedia Appendix 1 [file formative_v10i1e81259_app1.docx]

*Kai*- Food

*Karakia*– Prayer

*Koha*– A gift

*Kōrero*- Discussion

*Māori*- the Indigenous people of Aotearoa New Zealand

*Mahi*– Work

*Rangatahi*– Young people

*Te reo Māori*- the Māori language

*Whakawhanaungatanga*– Relationship building

*Whakaaro*– Thoughts and views
